# Antibiotic Resistance and Genotypes of *Helicobacter pylori* Strains in Patients with Gastroduodenal Disease in Southeast Poland

**DOI:** 10.3390/jcm8071071

**Published:** 2019-07-21

**Authors:** Izabela Korona-Glowniak, Halina Cichoz-Lach, Radoslaw Siwiec, Sylwia Andrzejczuk, Andrzej Glowniak, Przemyslaw Matras, Anna Malm

**Affiliations:** 1 Department of Pharmaceutical Microbiology with Laboratory for Microbiological Diagnostics, Medical University of Lublin, Chodzki Str. 1, 20-093 Lublin, Poland; 2 Department of Gastroenterology with Endoscopic Unit, Medical University of Lublin, Jaczewskiego Str. 8, 20-090 Lublin, Poland; 3 Department of Cardiology, Medical University of Lublin, 20-090 Lublin, Jaczewskiego Str. 8, 20-090 Lublin, Poland; 4 Chair and Department of General and Transplant Surgery and Nutritional Treatment, Medical University of Lublin, Jaczewskiego Str. 8, 20-090 Lublin, Poland

**Keywords:** pylorus infection, *cagA*, *vacA*, *iceA*, antibiotic patterns

## Abstract

The aim of this study was to investigate genetic diversity of *Helicobacter pylori* virulence markers to predict clinical outcome as well as to determine an antibiotic susceptibility of *H. pylori* strains in Poland. Gastric biopsies from 132 patients with gastrointestinal disorders were tested for presence of *H. pylori* with the use of rapid urease test, microbial culture, and polymerase chain reaction (PCR) detection. The genetic diversity of 62 *H. pylori* positive samples was evaluated by detection of *cagA* and PCR-typing of *vacA* and *iceA* virulence-associated genes. Most common *H. pylori* genotypes were *cagA*(+)*vacAs1m2* (27.4%) and *cagA*(−)*vacAs2m2* (24.2%). In logistic regression analysis, we recognized the subsequent significant associations: gastritis with *ureC*, i.e., *H. pylori* infection (*p* = 0.006), BMI index (*p* = 0.032); and negatively with *iceA1* (*p* = 0.049) and peptic ulcer with *cagA* (*p* = 0.018). Thirty-five *H. pylori* strains were cultured and tested by E-test method showing that 49% of strains were resistant to at least one of the tested antibiotics. This is the first study that reports the high incidence and diversity of allelic combination of virulence genes in gastroduodenitis patients in Poland. Genotyping of *H. pylori* strains confirmed the involvement of *cagA* gene and *vacAs1m1* genotype in development and severity of gastric disorder.

## 1. Introduction

*Helicobacter pylori* is an etiological factor of the most frequent and persistent bacterial infection worldwide, which affects nearly half of the world’s population. *H. pylori* infections are more common in developing countries—up to 90% of population, whereas in developed countries—below 40% [1]. In northern Europe, about 30% of adults are infected, whereas in south and east Europe the prevalence of *H. pylori* is often higher than 50%. The highest prevalence was reported in Portugal, similarly to Poland, where 84% of adult population is infected with this bacteria, which is a serious epidemiological problem [2,3]. *H. pylori* is associated with gastric ulcer, peptic ulcer disease, gastric cancer, and mucosa-associated lymphoma, but about 70% of infected population are carriers who stay asymptomatic [4].

The clinical outcome of *H. pylori* infection was supposed to be linked to certain strains differing in virulence factors presence or subtypes [5]. The presence of the cytotoxin-associated gene A (*cagA*) and the most active (*s1m1*) form of the vacuolating toxin (*vacA*) are the most important features of the bacterium in relation with higher risk of gastric adenocarcinoma and its pre-malignant lesions [6,7,8,9]. *H. pylori* is genetically heterogeneous, suggesting a lack of its clonality. It results in every *H. pylori*-positive subject carrying a distinct strain. This is possibly an adaptation of *H. pylori* to the gastric conditions of its host, as well as to the distinct patterns of the host-mediated immune response to *H. pylori* infection [10].

Treatment failure of *H. pylori* infection is caused mainly by progressive antibiotic resistance among *H. pylori* strains and numerous studies have shown that the prevalence of *H. pylori* antibiotic resistance varies significantly between countries, and even between regions within the same country. Local surveillance of antibiotic resistance is warranted to guide clinicians in their choice of therapy [11]. In regions with low resistance rates to clarithromycin (less than 20%), a standard first line-therapy which contains clarithromycin is recommended. In regions with high resistance to clarithromycin (>20%), first-line therapy with bismuth salts is recommended and, if it is not available, sequential therapy (Proton Pump Inhibitor (PPI), amoxicillin for 5 days and PPI, clarithromycin, metronidazole, or tinidazole for the next 5 days) [3,12].

Numerous diagnostic assays for *H. pylori* detection are available: bacterial culture, a rapid urease test (RUT), a urea breath test, histology, polymerase chain reaction (PCR), serology, and stool antigen test [13]. All these techniques have their limitations. Here we have tried to compare three diagnostic methods—microbiological culture, rapid urease test (RUT), and detection of *ureC* gene by PCR in biopsies from patients with gastroduodenal disease. Moreover, the pathogenicity of *H. pylori* strains was evaluated by detection of cytotoxin associated gene A (*cagA*), and genotyping of vacuolating cytotoxin gene A (*vacA*) and *iceA* gene and correlated them with the clinical outcomes. Antibiotic resistance was assessed by *H. pylori* culture and antibiotic susceptibility testing to monitor effectiveness of recommended treatments.

## 2. Material and Methods

### 2.1. Patients

Gastric biopsies were collected from 132 patients with indication of endoscopy (78 women and 54 men, the mean age 57.3 years, SD 15.6, median 59 years, range 20–87 years) who attended the Department and Clinic of Gastroenterology with Endoscopic Unit of Medical University of Lublin, Poland in 2016. Gastric biopsies were evaluated according to updated Sydney System. Chronic gastritis was defined as increased number of lymphocytes and plasma cells in the lamina propria of gastric mucosa. The degree of inflammation was referred to as mild, moderate and severe. Activity of inflammation was determined on the basis of neutrophilic infiltrates of the lamina propria, pits, or surface epithelium. Mild activity was diagnosed when less than one third of pits and surface was infiltrated, moderate—one-third to two-thirds, more than two-thirds defined severe activity of gastritis. The presence of atrophy, intestinal metaplasia, and intraepithelial neoplasia were determined. Attention was paid to the presence of lymphoplasia and erosions. In 23 male and in 21 female patients, moderate chronic gastritis with moderate activity was diagnosed, 2 remaining women had severe chronic gastritis with moderate activity. In two men, intestinal metaplasia was diagnosed and in none of the patients atrophic gastritis was confirmed. The participants with unintentional weight loss within the previous 6 months, upper abdominal mass, dysphagia, previous gastric resection, bleeding tendency and pregnancy were excluded from the study. Two biopsies from the distal corpus and two biopsies from the antrum were obtained during the panendoscopy. The endoscopic examination findings were: gastritis/duodenitis, peptic ulcer (gastric or duodenal ulcer), GERD (gastroesophageal reflux disease), and normal (Table 1). From each patient, informed consent was obtained. The Ethical Committee of the Medical University of Lublin approved the study protocol (no. KE-0254/174/2014).

### 2.2. Culture, Identification, and DNA Extraction

Samples were tested for the presence of urease with the use of RUT—Rapid Urease Test (CLO test Kimberly-Clark) and placed in tubes with 0.9% NaCl solution and transported to the Department of Pharmaceutical Microbiology with Laboratory for Microbiological Diagnostics, Medical University of Lublin, Poland. Biopsies were smashed with the use of two microscope slides and spread onto Schaedler agar (BioMerieux, Marcy-l’Étoile, France) with 5% sheep blood plates and Schaedler agar with 5% sheep blood plates additionally supplemented with DENT (Oxoid), incubated for 3–7 days at 35 °C in microaerophilic (5–10% CO_2_, 80–90% N_2_, 5–10% O_2_, Generbag microaer, BioMerieux) conditions. *H. pylori* isolates were identified by colony morphology, Gram-staining and positive catalase (3% H_2_O_2_), urease and oxidase (BBL DrySlide Oxidase—Becton Dickinson, Franklin Lakes, NJ, USA) tests.

DNA of biopsy sample and bacterial isolates were extracted using QIAGEN QIAamp DNA Mini Kit (Qiagen, USA) according to the manufacturer’s instructions. The identification in biopsy samples and isolates were further confirmed as *H. pylori* using *ureC* gene amplification with specific primers [14]. Extracted DNA was frozen to −70 °C until its use.

### 2.3. Amplification Experiments and Gene Detection

Determination of the presence *cagA* and genotypes of the genes *vacA s, vacA m, iceA* was performed by PCR amplification of the target genes from genomic DNA, using previously described oligonucleotides and protocols [15,16]. PCR reactions were performed with the use of the REDTaq ReadyMix PCR Reaction Mix (Sigma-Aldrich, St. Louis, MO, USA), followed by electrophoresis in 1.5% agarose gel (Sigma-Aldrich, St. Louis, MO, USA).

### 2.4. Antibiotic Susceptibility Testing

The strains obtained during the 72-h cultivation were then suspended in the brain heart infusion broth (BHI, Becton Dickinson, Germany). Cell concentration was determined using a densitometer (BioMeriux). Bacterial suspensions with a density of three according to the McFarland scale, i.e., 10^8^ cells (CFU)/1 mL were used for susceptibility testing. In subsequent steps, the susceptibility of the strains to the antibiotics amoxicillin (AC), clarithromycin (CH), metronidazole (MZ), tetracycline (TC), levofloxacin (LE), and rifampicin (RI) was determined by the E-test method using E-test strips (AB Biodisc, Solna). The strips were placed on Mueller–Hinton agar supplemented with 5% horse blood and 20 mg/L β-NAD. The incubation was performed in microaerophilic conditions for 3 days in 35 °C. Resistance breakpoints of *H. pylori* were interpreted according to EUCAST (AC—0.125 mg/L; CL—0.5 mg/L; TC—1 mg/L; MZ—8 mg/L; RI—1 mg/L; LE—1 mg/L).

### 2.5. Statistical Analysis

Data processing and analysis were performed using STATISTICA 13 (StatSoft. Inc., Tulsa, OK, USA). Shapiro–Wilk test was applied to test normal distribution of continuous variables. The Student *t*-test or Mann–Whitney U-test or Kruskal–Wallis ANOVA analysis for independent variables were used as intergroup comparisons component. The distribution of discrete variables in groups were compared with Pearson’s Chi-square test or the Fisher’s exact test. Odds ratio (OR) and their 95% confidence intervals (CI) were calculated. Logistic regression models were fitted to identify risk factors associated with different clinical diagnoses (GAST, GERD, PUD) using *H. pylori* genotypes and demographic data (age, gender, BMI, place of residence, smoking) as independent factors. Statistical significance was set at *p* < 0.05. The following indexes were calculated for RUT samples and microbial culture in relation to PCR samples: sensitivity, specificity, positive predictive value (PPV), and negative predictive value (NPV).

## 3. Results

**Figure 1:**
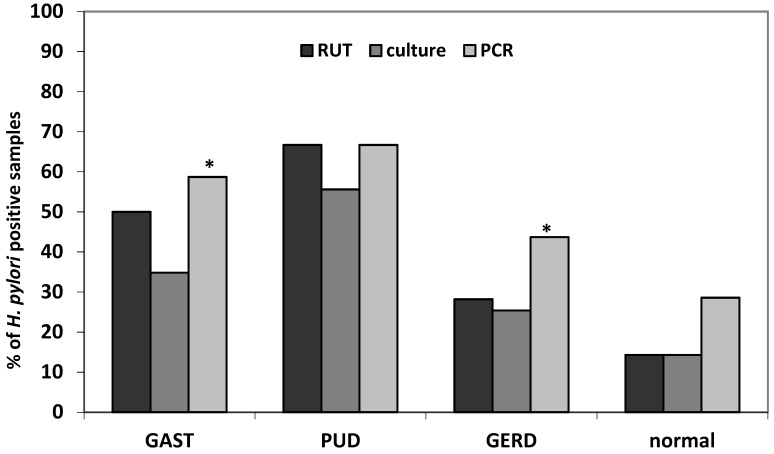
*H. pylori* identification using different methods regarding clinical diagnosis (GAST, gastritis/duodenitis; PUD, peptic ulcer disease (gastric/duodenal); GERD, gastroesophageal reflux disease); * *p* < 0.05 (PCR vs. culture).

Out of 132 patients, 35 (26.5%) microbial cultures, 45 (34.1%) RUT and 62 (47.0%) PCR tests were positive for *H. pylori*. Significantly higher detection rates for PCR method comparing to microbial culture (RR 3.6, 95%CI 2.6–4.95, *p* < 0.0001) and RUT (RR 11.6, 95%CI 3.8–34.7, *p* < 0.0001) were observed (Figure 1). Sensitivity of microbial culture in comparison to PCR identification was 56.5% and specificity 1.0% (PPV 100.0% and NPV 72.2%). RUT test sensitivity was 67.7% and specificity—95.7% (PPV 93.3% and NPV 77.0%). However, the same analysis carried out in different clinical outcomes showed significant difference in GAST and GERD groups only (Figure 1).

Table 1 presents demographic and clinical features of studied population. Male gender (57.4% vs. 39.7%; RR 1.4, 95%CI 1.0–2.0, *p* = 0.046) and residence in rural areas (66.0% vs. 36.9%; RR 1.5, 95%CI 1.2–2.0, *p* = 0.0019) were significantly associated with *H. pylori* infection.

Genotype distribution of *H. pylori* positive samples according to PCR analysis was presented in Table 2. It was shown that 56.5% of tested *H. pylori* were *cagA* positive. Regarding *iceA* gene: distribution of *iceA1* and *iceA2* were comparable (32.3% and 35.5%, respectively), and in 4.8% of samples mixed alleles *iceA1* + *iceA2* were detected. The *vacA s1m2* genotype was the most common in *H. pylori* positive patients, with 33.9%, followed by *s2m2* with 29.0% and *s1m1* with 19.4%. The co-infection of *s1m1* with *s1m2* and *s2m1* with *s2m2* was observed in 8% of *H. pylori* positive patients. All but two of the *vacA s1* alleles were *s1a* subtype (two strains had *s1b* subtype). None of *vacA m* alleles were detected in 4.8% of samples (*s1m0*).

The most common *H. pylori* genotype infecting studied patients were *cagA*(+) *vacA s1m2* (27.4%), *cagA*(−) *vacA s2m2* (24.2%) and *cagA*(+) *vacA s1m1* (19.4%). The prevalence of *cagA* gene as well as *vacA* and *iceA* genotypes varied in clinical outcome, yet without statistical significance (Table 3).

In univariate analysis, it was shown that *vacAs1m1* was significantly more frequent in patients with gastritis and peptic ulcer (Table A1). However, in logistic regression analysis including epidemiological factors as potential confounders, we recognized the subsequent significant associations: gastritis with *ureC* presence, meaning *H. pylori* infection (OR 3.3, 95%CI 1.4–7.9, *p* = 0.006), BMI index (OR 1.13, 95%CI 1.0–1.3, *p* = 0.032), and negatively with *iceA1* presence (OR 0.3, 95%CI 0.1–1.0, *p* = 0.049); peptic ulcer with *cagA* presence (OR 7.7, 95%CI 1.4–41.6, *p* = 0.018) whereas GERD with *iceA1* (OR 4.5, 95%CI 1.4–15.2, *p* = 0.014) and negatively with *cagA* (OR 0.32, 95%CI 0.1–0.9, *p* = 0.025).

Bacterial cultures were successfully isolated in 35 patients. MIC values of examined *H. pylori* strains showed that all of them were susceptible to amoxicillin and tetracycline (Table 4). Among all strains, 51% were susceptible to all and 49% were resistant to at least one of the tested antibiotics. Resistance to clarithromycin, metronidazole, levofloxacin, and rifampicin was found in 5 (14.3%), 11 (31.4%), 4 (11.4%), and 9 (25.7%), respectively.

**Figure 2:**
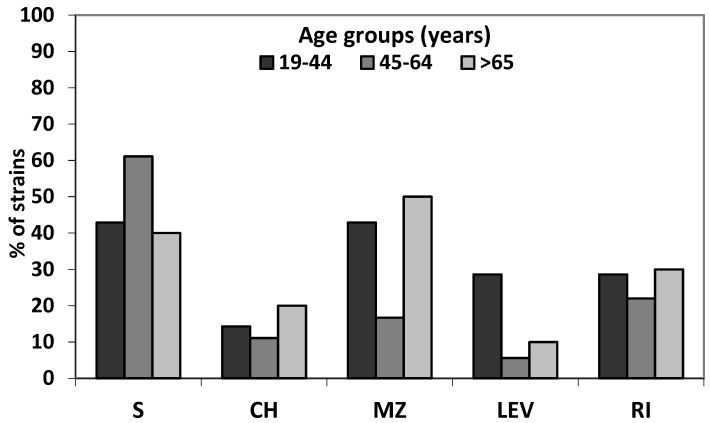
Resistance to tested antibiotics of 35 isolates *H. pylori* strains in different age groups (S—sensitive to all tested antibiotics; CH—clarithromycin; MZ—metronidazole; RI—rifampicin; LEV—levofloxacin).

Even though there was a slight difference in amount of resistant strains isolated from patients from different age group, showing their higher frequency in the oldest group, statistical analysis revealed no significant differences (*p* = 0.13) in distribution of resistance patterns between age groups (Figure 2).

The multidrug resistant strains (for two or more antibiotics) were observed in eight strains (22.9%). They were resistant to metronidazole and clarithromycin (50%) and/or levofloxacin (50%) and/or rifampicin (50%) (Table 5). Most of metronidazole resistant strains were found with high MIC value.

## 4. Discussion

There are several diagnostic assays for *H. pylori* infection, including bacterial culture, a rapid urease test (RUT), a urea breath test (UBT), histology, PCR, serology, and stool antigen test [10,11]. All these techniques have their limitations. The sensitivity of isolation of the bacterium has been reported to vary greatly among laboratories because of their fastidious nature [13]. Even the experienced laboratories recover the organism from only 50% to 70% of infected biopsies [17,18]. In this study, the low positivity rate of the culture (26.5%) may be due to a low number of bacterial cells, presence of non-culturable coccoid forms, contamination by other bacteria suppressing the growth of *H. pylori* or antibiotic therapy. Moreover, to avoid false negative results, it is recommended for patients not to consume the proton pump inhibitors (PPIs) two weeks before endoscopy, because these drugs indirectly interfere with *H. pylori* distribution in the stomach [19]. In our study, PCR was the most sensitive and specific method for detection of *H. pylori*. As the bacterial DNA was recovered from 56.5% of biopsies we consider this method as a reference. Advantages of PCR method are that it detects *H. pylori* in both forms i.e., spiral or coccoid forms that cannot be detected by other conventional methods [20], is able to discriminate the re-infection or recrudescence by genotyping of infective strains, enables to target pathogenic genes and assess the virulence potential of *H. pylori* in particular patient and does not require strict transport conditions. Therefore, PCR based methods are best for the specimens collected by invasive methods if the tests are carried out without contaminations. Nested PCR was proposed as a gold standard of *H. pylori* detection [13].

*H. pylori* infected patients develop superficial gastritis but the course of disease is partly due to environmental conditions and genetics of the host, but also due to the presence of particular virulence factors presented in the infecting *H. pylori* strains [21]. In this study, we focused on research of population and epidemiology of *H. pylori*, which provide us an information about genotypes linking them with clinical diagnosis. To our knowledge, this study presents the largest characteristic to date of *H. pylori* isolates collected from patients from the south east Poland. Distribution and relation between *H. pylori* genotypes and clinical outcomes, if derived from the different geographic regions may differ widely, then it is important to determine the most prevalent genotypes in a specific region [22].

An important application of standard PCR is the detection of specific pathogenic factors of *H. pylori*. There are two main pathogenic segments: the *cag*PAI and the polymorphism of the *vacA* gene. Other genes coding molecules associated with induction of inflammation (*dupA, iceA*) can also be detected by PCR. It was reported that *cag*PAI do cause more severe peptic ulceration, extra digestive diseases, and is associated with development of precancerous lesions and gastric adenocarcinoma [19]. In our study, in 56% of *H. pylori* positive samples *cagA* gene were found that was comparable to the other Polish studies [23,24], and logistic regression revealed relationship between *cagA* gene and clinical outcome. It was a risk factor positively associated with peptic ulcer diagnosis but negative association existed with GERD. Colonization with cagA-positive *H. pylori* strains was already shown to be inversely associated with reflux esophagitis and Barrett’s esophagus [25].

*H. pylori* also produces a vacuolating cytotoxin, VacA, which has been associated with the more severe diseases, e.g., peptic ulcer disease and gastric adenocarcinoma [26,27]. The gene encoding this cytotoxin is present in all strains but exhibits a mosaicism in the terminal (s) and median (m) regions. There are several alleles corresponding to various amounts of toxin produced: *s1m1* corresponds to the highest production, followed by *s1m2*, while strains with the *s2m2* allele do not produce any toxin. The *vacA* genotypes (allelic variations) are significantly different in each country and previous studies have confirmed important geographic differences in these virulence factors [27,28,29]. Among different genotypes, infection with *H. pylori* strains containing *vacA s1m1* type was strongly associated with increased risk of gastric cancer or PUD [8,30,31]. In our study, most frequent allelic combination of *vacA* gene were *s1am2*—38.7% and *s2m2*—32.3%, that was similar to previous Polish studies [23,24,32]; however, statistically significant associations between *vacAs1m1* type and GAST and PUD patients were observed (*p* = 0.042 and *p* = 0.038, respectively). Our findings also shown that among *cagA* positive strains, the combinations of *vacAs1m1/cagA+* was the predominant pattern in GAST and PUD patients. When both *cagA* and *vacA* detection is performed, a strong association exists between the presence of *cagA* and *vacA s1*, corresponding to strains with the highest production of cytotoxin [29,33] as well as more severe disease [34,35,36]. In our study, traditionally, less aggressive *vacAs2m2cagA(−)* were most common in GERD patients (32%).

Our laboratory findings suggest that gastric mucosa is colonized by mixed virulent type of *H. pylori* strains. In 8% of *H. pylori* positive samples mixed genotypes of *vacA* gene were detected, i.e., at least two different alleles of *m* region. Mixed *vacA* genotypes were also observed in other studies suggesting high probability of colonization by a few different *H. pylori* strains [28,29,33].

The role and correlation of *H. pylori iceA* (a gene induced by a contact with the gastric epithelium) in clinical outcomes is unclear. Our results revealed that 32.3% of *H. pylori* were harboring *iceA*1, 35.5% were *iceA*2, whereas 4.8% were mixed (*iceA*1/*iceA*2) which is consistent with other data from Poland but in younger population [24] and from Eastern Europe [37]. In other studies from European countries, it was shown that *iceA1* allele was two times more frequent than *iceA2* [15,38]. The *iceA1* allele, encoding a CATG-specific restriction endonuclease, is regulated by the contact of *H. pylori* with the human gastric cells, has been suggested to be related to PUD [15,16]. However, like the other authors, we doubt these findings [39,40]. In our study, *iceA1* was negatively associated with gastritis whereas it was positively associated with GERD. Further research of *H. pylori* strains with different *iceA* gene alleles could lead to clarifying its role in pathogenesis of human gastrodudenal diseases.

In our study, 35 *H. pylori* strains were tested; 51% of them were susceptible to all, and 49% were resistant to at least one of the tested antibiotics. Multiresistant strains accounted for as many as 23%, and included those resistant to two (metronidazole and clarithromycin or metronidazole and rifampicin or metronidazole and levofloxacin) or three (metronidazole, clarithromycin, and levofloxacin) antibiotics. This study showed lower primary antibiotic resistance rate than those reported in other areas of Poland or Europe [41,42]. In Eastern and Central European countries, the prevalence of *H. pylori* strains resistant to metronidazole is higher than in other developed countries, reaching almost 50%, and resistance to clarithromycin is as high as 30% and is still increasing, contributing to the failure of first-line therapy in approximately 70% of patients [32,41,42]. Such a high resistance to clarithromycin observed in Poland results mainly from excessive consumption of antibiotics, especially from the group of macrolides, and their widespread use in respiratory tract infections. Moreover, rates of resistance of *H. pylori* strains to levofloxacin have been very low in Poland [43], but the introduction of levofloxacin to eradication therapy of *H. pylori* infection quickly led to the emergence of resistant strains. Most of multi-resistant strains had genotype *cagA(+)* (75%) and *vacAs1* (62.5%).

A major limitation of our work was relatively low number of patients with specific clinical manifestations. We had only nine patients with peptic ulcer and none with gastric cancer, which can only be explained by limited number of studied patients, considering the published reports regarding its incidence in Poland [44]. We had also a very low number of *H. pylori* culture findings that might be related to absence of microorganisms in the gastric biopsy specimens, loss of viability during transport, and antibiotic or IPP intake.

Another limitation was that we studied only a few virulence genes although other genes involved in adherence (*babA*, *sabA*) or in pathogenesis (*oipA*, *dupA*) can be targeted to assess the virulence potential of *H. pylori.* To improve the significance of our study, especially the knowledge of populations from Eastern Europe, the importance of more recently disclosed *vacA* and *cagA* genotypes—such as *vacA* i-region/d-region and *cagA* EPIYA-region—would be worthy of investigation.

## 5. Conclusions

This is the first study that reports the high prevalence and diversity of allelic combination of *cagA, vacA*, and *icaA* genes in gastroduodenitis patients in southeast Poland. In our study, most frequent allelic combination of *vacA* gene were *s1m2* and *s2m2* and correlation between *vacAs1m1* type and development of GAST and PUD in patients was shown. Our findings also noted a lower primary antibiotic resistance rate than those reported in other areas of Poland. However, new antimicrobial resistance studies should be conducted periodically and regionally in our country to provide information that may help to monitor effective eradication programs.

## Figures and Tables

**Table 1:** Demographic and clinical features of studied population.

Clinical Diagnosis	No. of Patients (Mean Age in Years ± SD)		
	Male (*n* = 54)	Female (*n* = 78)	Total (*n* = 132)
Gastritis/duodenitis	23 (57.0 ± 15.3)	23 (57.5 ± 16.6)	46 (57.2 ± 15.6)
Peptic ulcer (gastric/duodenal)	4 (55.0 ± 12.6)	5 (66.4 ± 10.6)	9 (61.3 ± 12.3)
Gastroesophageal reflux disease	27 (58.1 ± 15.5)	44 (56.2 ± 16.4)	71 (56.9 ± 16.0)
Normal	1 (31.0)	5 (57.4 ± 17.6)	6 (53.0 ± 19.1)

**Table 2:** Distribution of *H. pylori vacA* genes/alleles according to *cagA* and *iceA* status.

*VacA* Alleles ^1^	*CagA* Status (%)		*IceA* Genotype (%)				Total (%)
	CagA(+)	CagA(−)	IceA1	IceA2	IceA1+2	Negative	
*s1m1*	11 (17.7)	1 (1.6)	4 (6.5)	5 (8.1)	0	3 (4.8)	12 (19.4)
*s1bm2*	1 (1.6)	0	0	0	1 (1.6)	0	1 (1.6)
*s1m1/s1m2*	1 (1.6)	2 (3.2)	0	2 (3.2)	1 (1.6)	0	3 (4.8)
*s1m2*	16 (25.8)	5 (8.1)	10 (16.1)	7 (11.3)	0	4 (6.5)	21 (33.9)
*s2m2*	3 (4.8)	15 (24.2)	5 (8.1)	5 (8.1)	0	8 (12.9)	18 (29.0)
*s2m1/s2m2*	0	2 (3.2)	0	1 (1.6)	0	1 (1.6)	2 (3.2)
*s2m1*	0	1 (1.6)	0	1 (1.6)	0	0	1 (1.6)
negative	1 (1.6)	0	0	0	0	1 (1.6)	1 (1.6)
Total	35 (56.5)	27 (43.5)	20 (32.3)	22 (35.5)	3 (4.8)	17 (27.4)	62 (100)

^1^ untyped m-region was found in three samples (*s1m0*).

**Table 3:** Genotypes of *H. pylori* strains and clinical outcome.

Genotype	GAST ^1^ (%)	PUD ^2^ (%)	GERD ^3^ (%)	*p-*Value
	*n* = 27	*n* = 6	*n* = 31	
*Vac1* genotypes ^4^				
*s1m1*	7 (25.9)	3 (50)	4 (12.9)	0.11
*s1m1/s1m2*	2 (7.4)	0	1 (3.2)	0.64
*s1m2*	9 (33.3)	3 (50)	11 (35.5)	0.74
*s2m2*	7 (25.9)	0	11 (35.5)	0.2
*s2m1/s2m2*	2 (7.4)	0	1 (3.2)	0.64
*s2m1*	0	0	1 (3.2)	-
negative	1 (3.7)	0	0	-
*cagA* status				
positive	16 (59.3)	5 (83.3)	16 (51.6)	0.35
negative	11 (40.7)	1 (16.7)	15 (48.4)	
*iceA* alleles				
*iceA*1	6 (22.2)	3 (50)	14 (45.2)	0.14
*iceA*2	10 (37.0)	3 (50)	8 (35.8)	0.42
*iceA*1+2	1 (3.7)	0	2 (6.5)	0.75
negative	10 (37.0)	0	7 (22.6)	0.14
*vacA s1m1/cagA+*	7 (25.9)	3 (50.0)	4 (12.9)	0.11
*vacAs1m2/cagA+*	6 (22.2)	2 (33.3)	9 (29.0)	0.78
*vacAs2m2/cagA+*	1 (3.7)	0	2 (6.5)	0.75
*vacAs1m1/cagA−*	1 (3.7)	0	1 (3.2)	0.89
*vacAs1m2/cagA−*	4 (14.8)	1 (16.7)	2 (6.5)	0.53
*vacAs2m2/cagA−*	6 (22.2)	0	10 (32.3)	0.23

^1^ GAST—gastritis/duodenitis; ^2^ PUD—peptic ulcer disease (gastric/duodenal); ^3^ GERD—gastroesophageal reflux disease; ^4^ untyped m-region was found in three samples (*s1m0*).

**Table 4:** Susceptibility and MIC values for 35 isolated *H. pylori* strains.

Antibiotics	Susceptibility (%)	MIC Range	MIC_50_	MIC_90_
Amoxicillin	35 (100)	<0.016	<0.016	<0.016
Clarithromycin	30 (85.7)	<0.016–>256	<0.016	16
Metronidazole	24 (68.6)	<0.016–>256	0.064	>256
Tetracycline	35 (100)	<0.016–0.19	<0.016	0.094
Levofloxacin	31 (88.6)	<0.002–>32	0.047	8
Rifampicin	26 (74.3)	<0.002–>256	0.5	2

MIC, Minimal Inhibitory Concentration; MIC_50_, MIC_90_, lowest concentration of antibiotic at which 50 and 90% of the isolates were inhibited.

**Table 5:** Characteristics of multidrug resistant *H. pylori* strains.

Age	Gender	Diagnosis	Genotype	Resistance Pattern	MIC (mg/L)					
					AC ^4^	CH ^5^	MZ ^6^	LE ^7^	RI ^8^	TC ^9^
53	M	GERD ^1^	*cagA(+)s1m1 iceA2*	CH+MZ+LE	<0.016	12	24	>32	0.125	0.19
64	F	GAST ^2^	*cagA(+)s1m1 iceA2*	CH+MZ	<0.016	>256	>256	0.047	0.75	<0.016
70	F	GERD	*cagA(−)s2m2 iceA2*	CH+MZ+LE+RI	<0.016	8	64	8	2	<0.016
71	F	GERD	*cagA(+)s1m2 iceA1*	MZ+RI	<0.016	<0.016	>256	0.023	>256	<0.016
44	M	PUD ^3^	*cagA(+)s1m2 iceA1*	MZ+RI	<0.016	<0.016	>256	0.125	2	<0.016
34	F	GERD	*cagA(+)s2m2 iceA1*	MZ+LE	<0.016	<0.016	>256	>32	<0.002	<0.016
29	F	GERD	*cagA(+)s1m2 iceA2*	CH+MZ+LE	<0.016	>256	>256	>32	0.5	0.094
73	F	GAST	*cagA(−)s2m2 iceA2*	MZ+RI	<0.016	<0.016	>256	0.047	2	0.19

^1^ GAST—gastritis/duodenitis; ^2^ PUD—peptic ulcer (gastric/duodenal); ^3^ GERD—gastroesophageal reflux disease; ^4^ AC—amoxicillin; ^5^ CH—clarithromycin; ^6^ MZ—metronidazole; ^7^ LE—levofloxacin; ^8^ RI—rifampicin; ^9^ TC—tetracycline.

## Appendix A

**Table A1:** Univariate analysis of factors associated with clinical diagnosis

Factor	GAST				PUD				GERD			
	GAST ^2^ (*n* = 46)	Others (*n* = 86)	OR (95%CI)	*p* Value	PUD ^3^ (*n* = 9)	Others (*n* = 123)	OR (95%CI)	*p-*Value	GERD ^4^ (*n* = 71)	Others (*n* = 61)	OR (95%CI)	*p-*Value
Age (mean ± SD)	57.2 ± 15.8	57.4 ± 15.7	1.0 (0.97–1.0)	0.81	61.3 ± 12.3	57.1 ± 15.8	1.0 (0.97–1.1)	0.43	56.9 ± 16.0	57.9 ± 15.3	1.0 (0.97–1.0)	0.72
Male gender	23 (50.0)	31 (36.1)	1.9 (0.9–3.9)	0.88	4 (44.4)	50 (40.7)	1.2 (0.3–4.5)	0.82	27 (38.0)	27 (44.3)	0.8 (0.4–1.6)	0.47
BMI ^1^ (mean ± SD)	27.0 ± 3.8	25.8 ± 3.4	1.1 (1.0–1.2)	0.06	27.2 ± 3.8	26.1 ± 3.6	1.1 (0.9–1.3)	0.42	25.9 ± 3.5	26.6 ± 3.8	0.9 (0.8–1.0)	0.22
rural residence	20 (42.5)	27 (31.8)	1.7 (0.8–3.5)	0.18	3 (33.3)	44 (35.8)	1.1 (0.2–4.7)	0.92	24 (33.8)	23 (38.3)	0.8 (0.4–1.7)	0.59
Smoking	12 (26.1)	28 (32.9)	0.7 (0.3–1.6)	0.42	3 (33.3)	37 (30.1)	1.4 (0.3–6.1)	0.66	20 (28.2)	20 (33.3)	0.8 (0.4–1.7)	0.52
*ureC*	27 (58.7)	35 (40.7)	1.9 (0.9–4.0)	0.075	6 (66.7)	56 (45.5)	2.4 (0.6–10.0)	0.23	31 (43.7)	31 (50.8)	0.8 (0.4–1.5)	0.41
*cagA*	16 (34.8)	19 (22.1)	2.0 (0.9–4.4)	0.093	5 (55.6)	30 (24.4)	3.9 (1.0–15.3)	0.054	16 (22.5)	19 (31.2)	0.6 (0.3–1.4)	0.27
*vacA s1m2*	10 (21.7)	13 (15.1)	2.0 (0.7–5.2)	0.17	3 (33.3)	20 (16.3)	3.4 (0.6–18.1)	0.15	11 (15.5)	12 (19.7)	0.7 (0.3–1.8)	0.48
*vacA s1m1*	8 (17.4)	6 (7.0)	3.4 (1.0–11.0)	0.042	3 (33.3)	11 (8.9)	6.2 (1.1–34.6)	0.038	5 (7.0)	9 (14.8)	0.4 (0.1–1.4)	0.17
*vacA s2m2*	7 (15.2)	13 (15.2)	1.4 (0.5–3.9)	0.56	0 (0)	20 (16.3)	-	-	12 (16.9)	8 (13.1)	1.2 (0.4–3.2)	0.77
*iceA1*	7 (15.2)	16 (18.6)	0.8 (0.3–2.1)	0.63	3 (33.3)	20 (16.3)	2.6 (0.6–11.1)	0.21	16 (22.5)	7 (11.5)	2.2 (0.9–5.9)	0.1
*iceA2*	11 (23.9)	14 (16.3)	1.6 (0.7–3.9)	0.29	3 (33.3)	22 (17.9)	2.3 (0.5–9.8)	0.26	10 (14.1)	15 (24.6)	0.5 (0.2–1.2)	0.13

^1^ BMI—body mass index; ^2^ GAST—gastritis/duodenitis; ^3^ PUD—peptic ulcer disease (gastric/duodenal); ^4^ GERD—gastroesophageal reflux disease.
